# EpiFire: An open source C++ library and application for contact network epidemiology

**DOI:** 10.1186/1471-2105-13-76

**Published:** 2012-05-04

**Authors:** Thomas Hladish, Eugene Melamud, Luis Alberto Barrera, Alison Galvani, Lauren Ancel Meyers

**Affiliations:** 1 Section of Integrative Biology, University of Texas at Austin, 1 University Station C0930, Austin, TX, 78712, USA; 2 Lewis-Sigler Institute for Integrative Genomics and Department of Chemistry, Princeton University, Princeton, NJ, 08544, USA; 3 Harvard Medical School, 77 Avenue Louis Pasteur, Boston, MA, 02115, USA; 4 Department of Epidemiology and Public Health, Yale University Medical School, New Haven, CT, 06520, USA; 5 Santa Fe Institute, 1399 Hyde Park Road, Santa Fe, NM, 87501, USA

## Abstract

**Background:**

Contact network models have become increasingly common in epidemiology, but we lack a flexible programming framework for the generation and analysis of epidemiological contact networks and for the simulation of disease transmission through such networks.

**Results:**

Here we present EpiFire, an applications programming interface and graphical user interface implemented in C++, which includes a fast and efficient library for generating, analyzing and manipulating networks. Network-based percolation and chain-binomial simulations of susceptible-infected-recovered disease transmission, as well as traditional non-network mass-action simulations, can be performed using EpiFire.

**Conclusions:**

EpiFire provides an open-source programming interface for the rapid development of network models with a focus in contact network epidemiology. EpiFire also provides a point-and-click interface for generating networks, conducting epidemic simulations, and creating figures. This interface is particularly useful as a pedagogical tool.

## Background

Epidemiological models traditionally assume mass-action dynamics: individual hosts in a population have identical contact rates and are well-mixed, such that any given pair may interact and transmit disease with equal probability [1,2]. Compartmental susceptible-infected-recovered (SIR) models implicitly assume mass-action interactions for infinite populations. However, the mass-action assumption is unlikely to be strictly valid in most instances. Although there are some settings in which mass-action models provide reasonable approximations, there are others in which it is essential to consider the heterogeneous contact patterns that underlie disease transmission [3,4].

Contact network epidemiology [5,6] explicitly models disease transmission through populations with heterogeneous contact patterns. Host populations are represented as networks of individuals (the nodes) and the contacts through which disease can spread (the edges between nodes). The definition of a disease-causing contact depends on the disease. For influenza, edges represent the potential for droplet or contact transmission, e.g., direct interactions such as prolonged proximity or food sharing [7], or indirect transmission of fomites persisting in the environment [8]. A node's degree is the number of contacts an individual has, and is one indication of an individual’s epidemiological importance, relative to other individuals in the population. The degree distribution of a network can play a critical role in shaping epidemic dynamics [4]. By modeling disease transmission probabilistically, contact network models can predict the expected epidemic size, the likelihood that an epidemic will occur given an introduction, and with some methods, the dynamics of an outbreak and likely chains of transmission through the population [9].

The 2003 SARS outbreak in China illustrates one limitation of mass-action models. Early estimates of the basic reproduction number (*R*_0_) for SARS predicted that 120 days after the disease was introduced in China, between 30,000 and 10 million individuals would have been infected in China [4]. In fact, only 792 SARS cases were reported [10]. This discrepancy stems, at least in part, from the assumption that disease transmission patterns for the entire Chinese population would be similar to the transmission patterns in the apartment building in Hong Kong and hospital in Singapore from which the early estimates were made. Such heterogeneity in contact patterns lends itself to a network approach, in which nodes can be connected to explicitly represent the diverse patterns of interactions that occur within human populations.

The spread of sexually transmitted infections is also often modeled more effectively with contact networks than mass-action models, because of non-randomness and heterogeneity in sexual contact patterns. An infected individual with many contacts may be more likely to spark a significant outbreak than an infected individual with fewer contacts. A study of 2,810 randomly sampled Swedes aged 18-74 years found that the number of sexual interactions per person approximately followed a power-law distribution, indicating significant heterogeneity in sexual activity [11]. A smaller study of sexual interactions among adolescents in a Midwest US town revealed long chains of contacts and fewer cycles (edges forming polygons) than would be expected by chance, patterns that can be readily captured by network models [12].

Contact network models also allow straightforward analyses of epidemiological dynamics and intervention events occurring at particular nodes. For example, the approach has been used to optimize the distribution of limited resources such as vaccines and antiviral drugs within a population [13-15], and to identify critical bridge groups connecting relatively disjoint parts of a high-risk population [16].

Although there are powerful mathematical methods for analytically estimating the dynamics of epidemics in complex contact networks [5,6], simulations of disease transmission through networks are also critical to the field. They allow us to test the validity of mathematical approximations and serve as the primary modeling approach when mathematical approximations cannot adequately describe the complexity of a population. There are two widely-used approaches to simulating disease transmission through networks: the Reed-Frost chain-binomial model [17,18] and the percolation model [19]. Although they can be made arbitrarily complex, these models typically represent individual hosts as nodes with discrete disease states, and propagate infection along edges from infected to susceptible nodes according to the probability of disease transmission, called transmissibility, which may either be fixed or determined by a function.

In the chain-binomial model, time is measured in arbitrary, discrete units. Simulations are parameterized with the number of time steps individuals remain infected, and the per-time-step transmissibility. The chain-binomial model may be used to generate epidemic curves (i.e., incidence time series data), identify chains of transmission, estimate the probability of an epidemic, and assess the distribution of epidemic sizes.

Percolation simulations give each infected individual one opportunity to spread disease to each of their susceptible contacts, but do so in an arbitrary, non-chronological order. One approach is to use a probabilistic breadth-first traversal of the network. In the simplest case, transmissibility is the same for all edges. One approach is to maintain four dynamic lists of nodes: (a) susceptible, (b) newly-infected, (c) currently-infected, and (d) recovered. At the beginning of a typical simulation, one or more nodes will be placed on the currently-infected list, and the remaining nodes will be on the susceptible list. Then the following two-step procedure is repeated until the currently-infected list is empty.

(1) For each currently-infected node, a uniform random number between zero and one is generated for each edge that connects the node to a susceptible neighbor. If and only if the random number for a given edge is less than the transmissibility, then the susceptible neighbor moves from the susceptible list to the newly-infected list.

(2) After all currently-infected nodes have been tested in this way for transmission, the nodes in the currently-infected list are moved to the recovered list and the nodes in the newly-infected list are moved to the currently-infected list.

A breadth-first approach like this will result in a coarse approximation of the epidemic curve, similar to a chain-binomial simulation with an infectious period of one unit. Percolation simulations tend not to predict realistic chains of transmission, since new infections occur in cohorts, and the order of transmission events is based on arbitrary orderings of each cohort. The percolation algorithm above can be modified slightly to simulate a chain-binomial model: rather than testing each edge from an infected to a susceptible node once, we test each edge *n* times, where *n* is the length of the infectious period or the number of time steps until transmission occurs, whichever occurs first. If parameterized appropriately, the two models will yield the same epidemic probability and final size distribution. The chain binomial model yields smoother, more realistic epidemic curves, while the percolation model is more computationally efficient.

While the field of contact network epidemiology is growing rapidly, it still lacks a flexible, user-friendly programming toolkit for generating contact networks, analyzing their structure and simulating the spread of disease through them. There are a few freely-available libraries for simulating and analyzing networks, but they are suboptimal for epidemiological research, particularly for novice programmers. Specifically, NetworkX [20], implemented in Python, is straightforward but slow, whereas igraph [21], implemented in C, is faster but less user-friendly. The R package statnet [22] is more specialized, focusing on statistical analysis of exponential-family random graphs. None of these packages provides epidemiological simulations or functions for calculating important epidemiological values. Other software packages provide valuable disease- or population-specific simulators (e.g., for pertussis [23], HIV [24], influenza [25,26], urban populations [27], metapopulation networks [28]), but lack a flexible framework for users to define alternative disease models and population structures.

Here, we introduce EpiFire, an applications programming interface (API) implemented in C++. EpiFire includes a fast and efficient library for generating networks with a specified degree distribution, measuring fundamental network characteristics, and performing percolation and chain-binomial simulations of SIR (susceptible-infected-recovered) disease transmission on generated networks. We have also developed a user-friendly interface that allows the user to perform these functions in a point-and-click environment and provides intuitive graphical results of epidemic simulations.

Although network models can be made to approximate mass-action models by assuming a completely connected network [29], it typically does not make sense to do so. Mass-action models are computationally very efficient and network models become computationally more demanding as the number of nodes and edges increases. Thus, EpiFire also includes a continuous time, stochastic mass-action simulation class to allow users to create hybrid models or to compare the results of mass-action and network-based models.

## Implementation

EpiFire comprises two bodies of code that are written in object-oriented C++: the applications programming interface (API) and the graphical user interface (GUI). The EpiFire GUI was developed using the API and Qt [30], and allows non-programmers to generate networks, perform epidemic simulations, and export figures and data. We describe the EpiFire GUI in more detail in the Results section below. The entire EpiFire code base is open source, licensed under GNU GPLv3.

The EpiFire API consists of 20 classes and 2,500 lines of non-whitespace code. The EpiFire GUI consists of 12 classes and 3,500 lines of non-whitespace code.

### Installation

EpiFire source code is available from GitHub at http://github.com/tjhladish/EpiFire/ or http://epifire.com. Users who have installed Git version control software (open-source, available at http://git-scm.com/) may create a local copy of the EpiFire repository by executing, without quotes, "git clone git://github.com/tjhladish/EpiFire.git" on the command line. Microsoft Windows and Mac OS X users can download precompiled binaries from http://sourceforge.net/projects/epifire/.

### EpiFire API

Functionally, the EpiFire API consists of tools for network generation, network manipulation, network characterization, and epidemic simulation. Programmatically, the API is divided into network, node, edge, and simulation classes. Each class defines a type of variable and its associated attributes and functions. For example, the network class allows users to define a network variable, which can contain one or more node variables that can be connected by one or more edge variables. In the small program below, an undirected network called my_network is created, and then populated with 100 nodes. The nodes are randomly connected with edges such that on average, each node will be connected to five others. Finally, the structure of the network is written out as an edgelist in the comma-separated-value format.

#include < Network.h>

int main() {

Network my_network("example network", Network::Undirected );

my_network.populate(100);

my_network.rand_connect_poisson(5);

my_network.write_edgelist("output.csv");

return 0;

}

The network constructor takes two arguments: an arbitrary text string naming the network, and either Network::Undirected or Network::Directed, which specifies whether all edges are undirected, or some or all may be directional.

Each time the program is run, a different randomly connected graph will be produced. The following is an example of the beginning of the output file:

0,97

0,17

0,21

1

2,49

2,51

2,36

2,73

2,66

3,45

In this case, Node 0 was connected to Nodes 97, 17, and 21. Node 1 was not connected to any others.

More sophisticated examples, including networks being used in epidemic simulations, can be found in Additional file 1 in the examples directory provided with the source code.

The network modeling portions of the code (the Network, Node, and Edge classes) can be used with or without the epidemiological code, and may therefore be useful for non-epidemiology applications. The simulation classes provided include three types of finite, stochastic epidemic simulations: percolation and chain-binomial (both network-based), and mass-action. Users may use the provided simulation classes or may create derived classes based on them. For example, the base class for percolation simulations, called Percolation_Sim assumes a disease with susceptible-infectious-recovered states. A simple derived simulation class can be created that inherits almost all the functionality of Percolation_Sim, but that uses an alternate progression of states. An example of a derived simulation using the susceptible-exposed-infectious-recovered state progression (SEIR_Percolation_Sim.h) can be found in the research directory provided with the source code.

Networks may be constructed explicitly by reading in an edgelist file, or adding individual nodes and specifying their connections. Networks can also be constructed implicitly by using one of the network generators provided. Generators for ring and square lattice networks are provided, as well as three random network generators: the Erdős-Rényi model [31], resulting in approximately Poisson degree distributions, the configuration model [32] that generates random networks with a user-specified degree distribution, 'and the Watts-Strogatz “small-world” network generation model [33].

Networks that are generated via the configuration model can contain edges that are usually undesirable in epidemiological models. Pairs of nodes may be randomly connected by two or more edges, and nodes may be “connected” to themselves by edges going to and from the same node. These edges, called parallel edges and self-loops respectively, may be removed using the provided “lose-loops” function (Additional file 1: Appendix B). This function uses a novel algorithm to reconnect the affected edges in a randomized way that preserves the degree sequence of the network. This approach may introduce some non-randomness to the network structure, but the improvement in algorithmic complexity over competing methods is significant [34].

Random numbers are generated using the Mersenne Twister algorithm [35] as implemented by Wagner, available at http://www-personal.umich.edu/~wagnerr/MersenneTwister.html.

### Percolation and chain-binomial pseudocode

EpiFire provides epidemic simulators using the percolation and chain-binomial models, represented as pseudocode below. Both pseudocode functions take a network as an argument and return final epidemic size. The most recent implementations, including additional functions for the simulators, can be found online [36,37]. In the percolation pseudocode below, *T* denotes the transmissibility of the pathogen, that is, the probability that transmission will occur between an infectious node and a susceptible neighbor.

**Percolation(*network*,*T*):**

*infected_queue* ← empty list

**foreach***node***in***network*:

set state of*node*to "susceptible"

*first_infected* ← random node from*network*

set state of*first_infected*to "infectious"

append*first_infected*to*infected_queue*

**while***infected_queue*is not empty:

*node* ← remove first element from*infected_queue*

**foreach***neighbor*of*node*:

*rand* ← uniform random number between 0 and 1

**if***neighbor*is "susceptible"**and***rand* < *T*:

set state of*neighbor*to "infectious"

append*neighbor*to*infected_queue*

set state of*node*to "recovered"

*epidemic_size* ← count of nodes in "recovered" state

**return***epidemic_size*

Appendix B2 of Additional file 1 provides a version of the percolation algorithm that produces an epidemic curve. In practice, it may be convenient to use integers as node states rather than text strings. In the chain binomial algorithm below, susceptible nodes have a value of 0, recovered nodes have a value of -1, and infectious nodes have a value equal to the number of days they have been infectious.

Appendix B3 of Additional file 1 provides a simple chain binomial function that performs one comparison per time unit per infectious node. Here, we describe a more efficient implementation. Instead of checking whether transmission occurs to each neighbor at each time step, we can determine the time until transmission along each edge. Because each transmission attempt can be considered a Bernoulli trial, we can determine when transmission will occur by sampling from a truncated geometric distribution with probability of "success" *T_cb* (chain binomial transmissibility) and support on {1, 2, . . . , gamma + 1}, where gamma is the infectious period. If the deviate happens to be gamma + 1, then transmission never occurs. In the pseudocode below, *transQ* is a priority queue of transmission events, sorted by time, least to greatest.

**Chain_binomial(*network*,*T_cb, gamma*):**

*transQ* ← empty priority queue of [time, node] pairs

*infected_list* ← empty list

**foreach***node***in***network*:

set state of*node*to 0

*current_time* ← 0

*first_infected* ← random node from*network*

Infect_node(*current_time*,*first_infected*)

**while***infected_list*is not empty:

**foreach***node***in***infected_list*:

increment state of*node*

**while***infected_list*is not empty:

**if**state of*infected_list*[0] ≤ *gamma*:

break

**else**:

set state of*infected_list*[0] to -1

remove first element from*infected_list*

**while***transQ*is not empty and time of*transQ*[0] ≤ *time*:

*event* ← transQ[0]

Infect_node(time of*event*, node of*event*,*T_cb*,*gamma*,*transQ*,*infected_list*)

*epidemic_size* ← count of nodes in -1 state

**return***epidemic_size*

**Infect_node(*current_time, node*,*T_cb*,*gamma, transQ, infected_list*):**

set state of*node*to 1

append*node*to*infected_list*

**foreach***neighbor*of*node*:

**if**state of*neighbor*is 0:

*rand* ← geometric_random_number(*T_cb*,*gamma*), see main text

**if***rand* ≤ *gamma*:

append [*current_time* + *rand*,*neighbor*] to*transQ*

### Analytic calculations of epidemic and network quantities

Given a degree distribution for a network and a transmissibility for a pathogen, the EpiFire API includes functions that calculate the expected epidemic threshold for the network (the critical transmissibility above which epidemics are possible), the basic reproductive rate of the pathogen in that network (*R*_*0*_). EpiFire GUI further calculates expected epidemic size under network and mass-action assumptions. All of the network calculations assume the configuration network model, such that the network is a random draw from all randomly connected networks with the specified degree distribution. Calculations, unless otherwise noted, are adapted from Meyers (2007) [6], which provides additional mathematical details.

The epidemic threshold for a network is a critical transmission probability (along edges) below which outbreaks are expected to fizzle out and above which large epidemics are possible, but not guaranteed. Technically, in an infinite network, outbreaks below the epidemic threshold will reach only a finite number of nodes, while outbreaks above the threshold can either be finite or infect a fraction of the network including an infinite number of nodes. This value is a function of the network structure and corresponds exactly to an *R*_0_ value of 1; given by

(1) $$T_{c}=\frac{\Sigma_{k}kp_{k}}{\Sigma_{k}k(k-1)p_{k}}\text{,}$$

where *k* is the degree of a node, and *p*_*k*_ is the fraction of nodes having degree *k*.

The expected basic reproductive rate is the expected number of neighbors that will be infected by each infectious node early in an epidemic, and is equal to the ratio of the actual transmissibility to the critical transmissibility, given by

(2) $$R_{0}=\frac{T}{T_{c}}\text{.}$$

The expected epidemic size is then given by

(3) $$E_{\mathrm{net}}=1-\Sigma_{k}p_{k}{\left(1+(u-1)T\right)}^{k}\text{,}$$

where *u* is the solution to the self-consistency equation

(4) $$u=\frac{\Sigma_{k}kp_{k}{\left(1+(u-1)T\right)}^{k-1}}{\Sigma_{k}kp_{k}}\text{.}$$

We also provide a function that calculates the expected final epidemic size in a mass-action model, given a value of *R*_0_[1]:

(5) $$E_{\mathrm{ma}}=S_{0}(1-e^{-R_{0}E_{\mathrm{ma}}})\text{,}$$

where *S*_0_ is the fraction of individuals who are susceptible at the start of the epidemic. The expected epidemic sizes under both the mass action and network models are solved numerically using the bisection method [38].

By calculating and comparing the network and mass-action expectations for an epidemic size of a specific network-pathogen combination (done automatically in the EpiFire GUI), one can assess the epidemiological impact of the network structure. Large differences in the values of network and mass-action expectations suggest that network structure plays an important role in disease transmission, and that traditional compartmental models may not be adequate.

Since percolation and chain binomial transmissibilities are per-time-unit and per-infectious-period probabilities, respectively, when users switch between simulation types the transmissibility parameter is recalculated accordingly.

One important property of networks is clustering, a measure of whether nodes exist in well-interconnected groups. EpiFire implements the transitivity clustering coefficient [39], calculated as

(6) $$Transitivity=\frac{3*triangles}{triples}\text{,}$$

where triangles is the number of sets of nodes A, B, and C such that all three are interconnected, and triples is the number of sets of nodes A’, B’, and C’ such that B’ is connected to A’ and C’.

## Results

EpiFire is an applications programming interface (API) implemented in C++, designed to efficiently generate networks with a specified degree distribution, measure fundamental network characteristics, and perform percolation and chain-binomial simulations of SIR disease transmission for generated networks. EpiFire also includes a continuous time, stochastic mass-action simulation class for creating hybrid models and/or comparing the results of mass-action and network-based simulations.

EpiFire allows users to develop efficient epidemic simulations in C++ by providing a high-level API for running simulations and manipulating the underlying contact networks in network-based models. The following examples demonstrate simple use-cases.

### Example 1: Percolation simulation (API)

This percolation simulation is performed using a random network constructed using the Erdős-Rényi algorithm with 10,000 nodes and mean degree 5. The probability of transmission between an infected node and a susceptible neighbor is 0.25, and the epidemic begins with 10 infected nodes (selected randomly without replacement).

#include < Percolation_Sim.h>

int main() {

// Construct Network

Network net("example net", Network::Undirected);

net.populate(10000);

net.fast_random_graph(5);

// Parameterize and run simulation

Percolation_Sim sim(&net);

sim.set_transmissibility(0.25);

sim.rand_infect(10);

cout < < "Expected R0: " < < sim.expected_R0() < < endl;

sim.run_simulation();

cout < < "Epidemic size: " < < sim.epidemic_size() < < endl;

}

Sample output:

Expected R0: 1.25551

Epidemic size: 3423

The output from this example is the expected value of *R*_0_ if an epidemic occurs (see Implementation) and the total number of individuals infected during the epidemic. By running the simulation many times, we can generate a distribution of epidemic sizes. Alternatively, to generate an epidemic curve, we can report the size of the infected cohort after each round of transmission (see Additional file 1: Appendix A).

Example 1 required 0.06 sec (avg) and 5.45 MB (max) of system memory. The test system was a Dell Precision Workstation 490 with two Intel Xeon 5140 processors and 4 GB of RAM running 32-bit Ubuntu 10.04 LTS. EpiFire was compiled using gcc version 4.4.3 with O2 optimization. Most of the time is spent constructing the random network; the simulation itself only requires 0.5 ms (avg). Depending on the intended application, it may be acceptable to generate and reuse a single network for many simulations, greatly reducing the time required. Users should note that when reusing a network, sim.reset() should be called in between simulations to reset the state of all nodes to the default susceptible state, as in Appendix A3 of Additional file 1. The running time required for Example 1 scales linearly with the expected epidemic size, whereas the memory required is linear with *N* * (*k* + 1) where N is the network size and k is the mean degree.

### Additional API examples

Appendix A2 of Additional file 1 includes a more complicated simulation of an epidemic on a dynamic network. Further examples included with the source code are a chain-binomial simulation of a network with an arbitrary degree distribution; a derived percolation class that uses a susceptible-exposed-infectious-recovered progression of states; and a stochastic, continuous time, mass-action simulation using a Gillespie algorithm [40,41].

### Comparison with NetworkX and igraph

EpiFire is not intended to replace other network APIs, which were developed to solve different problems. To compare these diverse APIs, we consider one of their common functions: generation of an Erdős-Rényi random network. There are several algorithms that will generate random networks; we chose the most efficient algorithm available in each API when generating a 100,000 node network with a Poisson degree distribution with Poisson parameter (mean) equal to ten. EpiFire requires much less memory and running time than the user-friendly NetworkX, and somewhat less memory and time than igraph (Table 1). The comparatively poor performance of NetworkX is likely due primarily to differences in efficiency between C++ and Python.

**Table 1:** Comparison with NetworkX and igraph

	Memory required (MB)	Time required (sec)
EpiFire API	45	0.830
NetworkX	1,400	15.4
Igraph	93	0.995

Performance comparison with NetworkX and igraph, generating random networks with 100,000 nodes, Poisson(10) degree distribution. Network generation in NetworkX was performed using the fast_gnp_random_graph() function.

### Overview of EpiFire GUI

Although the EpiFire API provides great flexibility for creating custom simulations, it requires some background in programming. As a demonstration of some of the capabilities of the EpiFire API, we present the EpiFire graphical user interface (GUI), which allows users to generate and analyze several common classes of random networks and conduct chain-binomial and percolation SIR simulations on contact networks in a point-and-click environment with intuitive, automatically generated figures. The EpiFire GUI requires no programming to create and analyze networks and run stochastic simulations on those networks.

#### Main window

**Figure 1:**
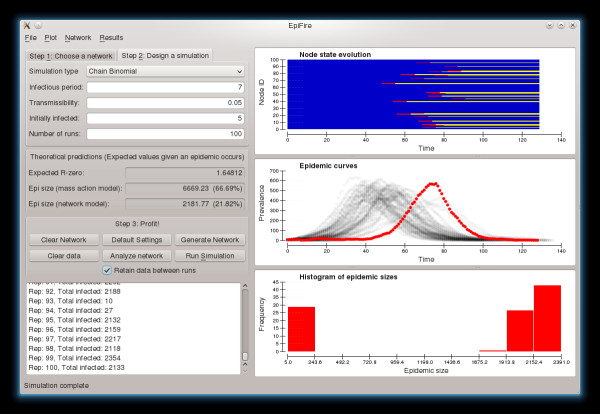
EpiFire GUI main application window.

The application's main window (Figure 1) is organized in two panes, with model parameters and application status on the left, and automatically generated plots of simulation data on the right. The left-hand pane is divided from top to bottom, as follows:

##### Network parameterization

By default, the tab labeled “Step 1: Choose a network” is active. Users choose whether to import a network from a file or to randomly generate a network. The import format is an edge-list file, with each edge represented as a single line containing the names of the connected nodes, separated by a comma. Currently only undirected networks are supported by the EpiFire GUI. If users choose to generate a network, they may specify the desired number of nodes, the degree distribution type, and relevant parameters for the degree distribution. Generated networks are connected randomly using the configuration model with the constraint that no pair of nodes is connected by more than one edge, and no edges loop back to connect a node to itself. Users may select Poisson, exponential, power law, urban, or fixed degree distributions. Degree distributions are right-truncated at *n* – 1, where *n* is the size of the network. Exponential and power law distributions are also left-truncated so that there are no nodes with degree zero. The urban degree distribution is a semi-empirical distribution used previously to study the spread of SARS and influenza in Vancouver, Canada [4,13,14].

##### Simulator parameterization

By clicking the tab labeled “Step 2: Design a simulation,” users may specify simulation parameters. Epidemics can be simulated under chain-binomial and percolation models. Chain-binomial is the default because it produces epidemic curves with finer temporal resolution, although percolation simulations run faster and will produce the same distribution of final epidemic sizes. Both simulators allow users to specify a transmissibility, the number of infections that should start the epidemic, and the number of simulation repetitions that should be performed. Chain-binomial simulations are also parameterized with an infectious period, defined as the number of time-steps an infected individual will remain infected; when this is set to 1, the chain-binomial and percolation models produce equivalent results. Users may also choose whether epidemic data is retained between runs or deleted prior to each new simulation. This determines which data are included in the plots.

##### Theoretical predictions

The EpiFire GUI also displays the expected *R*_0_ for the current network and epidemic simulation parameters. Epidemics will not occur when *R*_0_ is less than one, but may occur otherwise. Given this expected *R*_0_, EpiFire calculates expected epidemic sizes under mass-action and configuration model assumptions.

##### Control panel

The control panel allows users to clear the current network or the current epidemic data from memory, restore the default settings, open the help dialog, generate and load networks, and run a simulation with the specified parameters. Note that when “Generate Network” or “Import Edge List” is clicked, any previous network is automatically cleared, and the “Run Simulation” button is disabled unless a network has been created.

##### Status log

The status log provides users with updates, including the status of network generation, warnings about incompatible parameters, current simulation number, and final epidemic size.

The right pane of the EpiFire GUI is divided into three plots (initially blank) of simulation results. These plots may be resized by resizing the main window, or by clicking and dragging the horizontal dividers between the top and middle, and the middle and bottom plots. All of the plots created by the EpiFire GUI can be exported by double-clicking the plot, and the data used to generate the plots can be exported by right-clicking.

##### Node state plot

The top plot shows the progression of states of the first 100 nodes in the network, or all nodes if the network has fewer than 100 nodes. The horizontal axis represents the duration of the epidemic, and each horizontal band represents the states of a particular node. Blue denotes susceptible, red is infectious and yellow is recovered. The range of the horizontal axis is the total duration, in time-steps, of the most recent simulation run. These plots may provide visual insights into synchrony between node states and the relative amount of time nodes spend in each state.

##### Epidemic curve plot

The middle plot displays the number of individuals in the infectious state at each time step. The most recent epidemic curve (representing the most recent simulation run) is shown in red. If users choose to retain data between simulation runs, then all previous simulations are shown in semi-transparent gray. These gray data points effectively become a density plot, so that after many runs, users can see the range of possible outcomes and what a typical epidemic might look like.

##### Histogram of epidemic sizes

The bottom plot shows how many times epidemics of a given size class have been observed, where epidemic size is defined as the total number of nodes in the recovered state at the end of the epidemic (when there are no remaining infectious nodes). As more simulation runs are compiled, the histogram of observed epidemic sizes more accurately estimates the distribution of possible epidemic sizes.

#### Network visualization window

Displaying large networks is difficult, especially those with random connections that are uncorrelated with any two-dimensional location. If networks are small (< 100 nodes), it may, however, be useful to display their structure. We provide a “Show network plot” option within the “Plot” menu, which uses a variant of the Fruchterman-Reingold algorithm [42] to plot nodes and edges in a pop-up window. Our algorithm deviates from the classical Fruchterman-Reingold by preferentially placing high-degree nodes near the center of the plot, rather than starting with a uniform distribution of nodes. This plot is dynamic, allowing users to explore or improve the plot by clicking-and-dragging nodes to new locations. Users may zoom in and out using the +/- keys, respectively. The network plot option is disabled for networks with more than 500 nodes due to the complexity of the algorithm used.

#### Network analysis window

**Figure 2:**
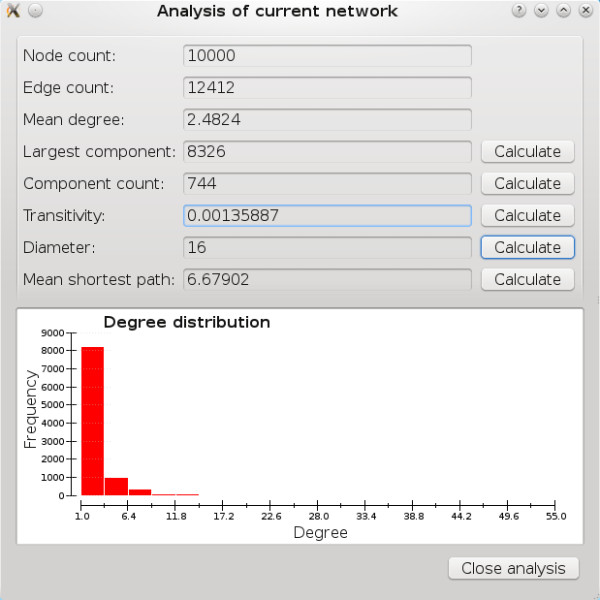
"Analysis of current network" dialog.

The “Network” menu includes a “Network analysis” option. If there is a network in memory, a new pop-up window (Figure 2) appears with the node count, edge count, mean degree, and a histogram of the degree distribution. By clicking on the “Calculate” buttons, the user can determine the number of nodes in the largest component, number of components, transitivity clustering coefficient, diameter of the largest component, and mean shortest path in the largest component. Note that the last two calculations are computationally demanding and can take much longer to complete than the others. In some cases, calculating one statistic involves first calculating another. In this situation, all calculated statistics will be shown, even if the user did not click “Calculate” for each of the statistics.

The network analysis window is particularly useful for comparing networks with different degree distributions, and for elucidating unexpected simulation results. For example, a simulation with a very high expected *R*_0_ may fail to create correspondingly large epidemics if the underlying network has multiple components.

#### Simulation results analysis window

**Figure 3:**
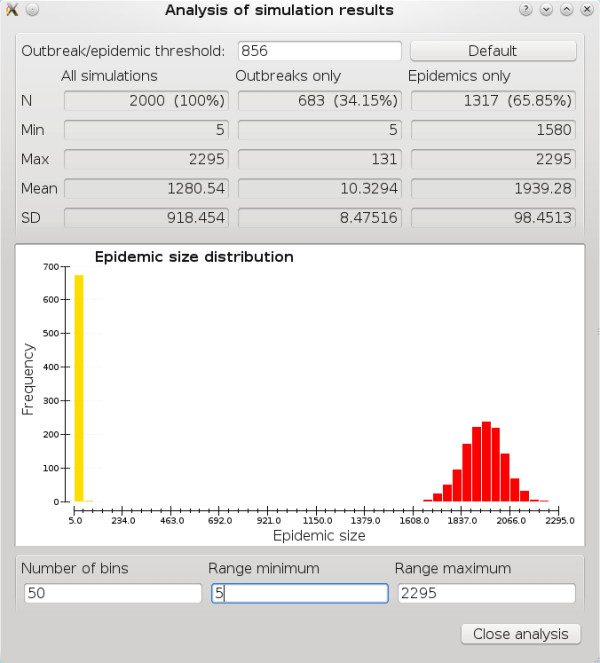
"Analysis of simulation results" dialog.

Under the “Results” menu is the “Simulation results analysis” option. Once results have been generated, users can open a new window (Figure 3) that automatically calculates basic statistics about the distribution of final epidemic sizes, including minimum, maximum, arithmetic mean, and standard deviation. Because final size distributions are commonly bimodal with the smaller mode corresponding to failed epidemics (called “outbreaks” in the analysis window) and the larger mode corresponding to actual epidemics, these statistics are also calculated separately for the two modes. The EpiFire GUI attempts to heuristically distinguish outbreaks from actual epidemics by checking to see if there is a single large range separating two clusters of data. If such a range exists, the middle of the range is used as the “outbreak/epidemic threshold,” which users may always change to a different value. The epidemic size distribution plot shows values below the threshold in yellow, and those equal to or above the threshold in red. Users may customize the plot by specifying the number of bins and the minimum and maximum values to use on the horizontal axis.

### Example 2: Percolation simulation (GUI)

The simulation in Example 1 can also be performed using the GUI according to the instructions below. Default settings are assumed unless indicated.

1. Click “Generate Network” or press CTRL-G to create a 10,000 node network with a Poisson degree distribution with expected mean degree equal to 5 (these are the default settings on the “Choose a network” tab).

2. Click the “Design a simulation” tab or press ALT-2

i. Change “Simulation type” to Percolation

ii. Change “Transmissibility” to 0.25

iii. Change “Initially infected” to 10

3. Click “Run Simulation” or press ENTER to run the simulation.

The epidemic size will be printed in the log window in the lower left. Figures characterizing the simulation run will be automatically generated on the right, including a node-state plot, an epidemic curve plot, and an epidemic size histogram. The epidemic size histogram will better approximate the true final size distribution as additional simulations are performed.

## Discussion

Developing epidemiological simulations that scale effectively to millions of individuals can be challenging. The open source API of EpiFire provides a transparent, logical framework that can be used for standard percolation, chain-binomial, or mass-action SIR simulations. Furthermore, it can be extended to create new, specialized types of simulations, such as networks that change in response to epidemic dynamics, or multi-pathogen simulations where co-infection changes transmission probabilities. EpiFire allows for hybridized models and alternative network interpretations, such as using a mass-action model for within-city dynamics and a network model for between-city dynamics [15].

Several other publically-available software projects have overlapping functionality. However, none have been written specifically for contact network epidemiology with the intent of providing a common, extensible toolkit for researchers to use to develop their own models.

Although EpiFire is intended as an API for contact network epidemiology, the network class is independent from the simulation classes, and is thus applicable to other types of network-based modeling, such as metabolite interaction networks [43] and animal migration between habitats [44].

The EpiFire graphical interface provides a user-friendly toolkit for performing network-based SIR epidemic simulations and gaining an intuitive understanding of the impact of network structure on infectious disease dynamics. The most obvious applications are pedagogical: the straight-forward interface and rapid feedback allow users to learn first-hand the consequences of changing epidemic and network parameters. EpiFire has been used in courses at the University of Texas at Austin and at the Summer Institute in Statistics and Modeling in Infectious Diseases (SISMID) at the University of Washington [45]. EpiFire GUI may be particularly useful to researchers during initial epidemiological explorations of a new contact network because of the ease with which it generates figures and network statistics.

We are currently adding support for deterministic, ordinary differential equation models, which will include derived classes implementing the standard mass-action SIR model, and a network-based SIR model [46,47]. The stochastic, continuous time mass-action model that EpiFire currently provides in MassAction_Sim.h will likely be refactored into a Gillespie model base class and mass-action and network derived classes. Finally, although the EpiFire simulators can be extended beyond SIR epidemic models (e.g., see [48] for the susceptible-exposed-infected-recovered simulator), we would like to provide a generic interface for specifying an arbitrary disease-state progression.

## Conclusions

Efficient and easy-to-use software plays a critical role in computational biology research. Contact network approaches in epidemiology provide sophisticated analytical and efficient computational methods, but these can be technically challenging and time consuming to implement. Currently no open-source toolkit is available for facilitating contact network epidemiology research. We present EpiFire, an applications programming and graphical interface, available for Windows, OS X, and Linux online at sourceforge.net/projects/epifire. As the field of contact network epidemiology matures, so should its mathematical and computational toolkit. Open-source code libraries like EpiFire help to avoid programming mistakes, increase the transparency of analyses, and reduce barriers between the conception and implementation of ideas.

## Availability and requirements

Project name: EpiFire

Project home page: https://github.com/tjhladish/EpiFire/wiki/ for source code, http://sourceforge.net/projects/epifire/ for binary installers

Operating systems: Platform independent

Programming language: C++

Other requirements: g++ 4.5 for API; g++ 4.5 and Qt 4.7 for GUI

License: GNU GPLv3

Any restrictions for use by non-academics: none

## Competing interests

The authors declare that they have no competing interests.

## Authors’ contributions

TH conceived of and implemented the project and drafted the manuscript. EM assisted in developing the programmatic and graphical interfaces. LAB developed a beta version of the graphical interface. LAM helped to implement analytical methods, refine the graphical interface and draft the manuscript. AG helped to draft the manuscript. All authors read and approved the final manuscript.

## Supplementary Material

Additional file 1 Appendices A and B. Click here for file
