# Supplementary material for: EpiFire: An open source C++ library and application for contact network epidemiology
Source: BMC Bioinformatics. 2012 May 4;13:76. doi: 10.1186/1471-2105-13-76 (PMC3496579; doi:10.1186/1471-2105-13-76)
Supplement: Additional file 1 — Appendices A and B. [file 1471-2105-13-76-S1.pdf]

Appendix A: More API examples

Example A1: Mass-action simulation

This example is a C++ program that uses a mass-action model to simulate the spread of a disease in a population with size 10,000. The parameters used describe a disease where the expected time to recovery after infection is 6 days, and the expected time between contacts between individuals is 4 days.

```
#include "MassActionSim.h"

int main() {

    int N        = 10000;    // Simulated population size
    double GAMMA = 1/ 6.0;   // Recovery rate
    double BETA  = 1/ 4.0;   // Contact rate

    MassActionSim sim(N,GAMMA,BETA);
    sim.rand_infect(1);            // Infect 1 individual
    sim.run_simulation();
    cout << sim.epidemic_size() << endl;  // Print final size

    return 0;
}
```

Mass-action models tend to be particularly fast and memory efficient. Running Example 2 10,000 times in a loop required 22.5 wallclock seconds (2.25 ms/simulation) and a maximum of 130 KB of RAM. These simulations scale approximately linearly with population size. When Example 2 was performed using a population size of 10 million and 100 initial infections (effectively guaranteeing an epidemic), each simulation required 13.13 sec and 100.3 MB of RAM.

Example A2: Percolation on a dynamic network

The following is a more complex program that simulates an epidemic on a network with a topology that is changing during the epidemic. Each epidemic cohort attempts to transmit disease on a static network, then the network is modified by allowing some nodes to re-form all new edges, followed by another round of disease transmission. For more detail, refer to the in-line comments, which are denoted by a double forward-slash (//).

```
#include <Percolation_Sim.h>

// This example simulates an epidemic on a changing network using an SIR
// percolation simulation.  Since networks that change with time can be
// implemented in many different ways, here is a more detailed description:
//
// During each infectious period, 10% of all nodes "migrate".  A different
// 10% is randomly selected each period.  If a nodes is in the migrating
// group, all of its edges are broken; this means the node's neighbors also
```

```
// end up with at least one broken edge.  These broken edges ("stubs") are
// then randomly reconnected.  The reconnection process will make sure that
// no self-loops or parallel edges get created (it's possible that
// additional edges will get shuffled in order to get rid of these).


int main() {

    // Construct an undirected network
    // with size 10,000 and a poisson(5)
    // degree distribution
    Network net("name", );
    net.populate(10000);
    net.fast_random_graph(5);

    // Create variables to handle node migration
    vector<Node*> nodes = net.get_nodes();
    vector<Edge*> broken_edges;
    double fraction_to_migrate = 0;
    int num_to_migrate = fraction_to_migrate * net.size();

    // Set up simulation
    Percolation_Sim sim(&net);
    sim.set_transmissibility(0.25); // Epidemic size will be 37-38%
    sim.rand_infect(10); // The probability of an epidemic will be ~99%

    // Continue the simulation as long as someone is still infected
    while (sim.count_infected() > 0) {
        sim.step_simulation(); // This advances the simulation by one
infectious pd

        shuffle(nodes, net.get_rng()); // Shuffle the nodes
        for (int i = 0; i < num_to_migrate; i++) { // Migrate the first 1000
            Node* node = nodes[i];
            vector<Edge*> edges_out = node->get_edges_out();
            vector<Edge*> edges_in = node->get_edges_in();

            // Break all outbound edges
            for (unsigned int j = 0; j < edges_out.size(); j++) {
                Edge* outbound_edge = edges_out[j];
                if (not outbound_edge->is_stub() ) {
                    edges_out[j]->break_end();
                    broken_edges.push_back(edges_out[j]);
                }
            }

            // Break all inbound edges
            for (unsigned int j = 0; j < edges_in.size(); j++) {
                Edge* inbound_edge = edges_in[j];
                if (not inbound_edge->is_stub() ) {
                    edges_in[j]->break_end();
                    broken_edges.push_back(edges_in[j]);
                }
            }
        }
        // Randomly reconnect all broken edges
        net.rand_connect_stubs(broken_edges);
```

```
        broken_edges.clear();
    }
    // The following line is not necessary, but validate() will check
    // to make sure that the network structure is still valid.  For example,
    // If Node A has an edge leading to Node B, Node B must know it has an
    // inbound edge from Node A.  In undirected graphs, there must be a
    // complementary edge from Node B to Node A.  (Technically, these are
arcs.)
    net.validate();

    // Print out the final epidemic size
    cout << sim.epidemic_size() << endl;
    return 0;
}
```

Example A3: Simulating epidemic curves

```
#include <Percolation_Sim.h>

int main() {

    // Construct Network
    Network net("name", false);
    net.populate(10000);
    net.sparse_random_graph(10);
    int reps = 10;

    for (int i = 0; i < reps; i++){
        // Choose and run simulation
        Percolation_Sim sim(&net);
        sim.set_transmissibility(0.25);
        sim.rand_infect(1);
        cout << "Rep " << i << "\n" << sim.count_infected() << ",";
        while (sim.count_infected() > 0) {
            sim.step_simulation();
            if (sim.count_infected() == 0) {
                cout << "0\n";
            } else {
                cout << sim.count_infected() << ",";
            }
        }

        cout << "Final size: " << sim.epidemic_size() << "\n\n";
        sim.reset();
    }
    return 0;
}
```

Sample output:

```
Rep 0
1,2,8,29,67,165,368,826,1614,2351,2042,984,357,105,23,6,2,1,0
Final size: 8951

Rep 1
```

1,2,5,10,22,56,131,282,674,1350,2187,2204,1295,447,135,37,9,4,2,1,0
Final size: 8854

Rep 2
1,3,11,32,85,211,495,1036,1859,2311,1739,776,258,77,17,1,2,1,0
Final size: 8915

Rep 3
1,7,11,21,60,152,338,805,1574,2265,2069,1071,375,115,33,8,3,1,0
Final size: 8909

Rep 4
1,2,1,0
Final size: 4

Rep 5
1,1,3,7,20,39,96,237,544,1121,1943,2259,1617,724,231,65,11,1,1,0
Final size: 8921

Rep 6
1,2,4,13,24,59,132,324,724,1384,2130,2168,1240,464,128,35,6,2,0
Final size: 8840

Rep 7
1,3,9,18,44,121,296,692,1390,2212,2167,1289,491,131,37,15,4,0
Final size: 8920

Rep 8
1,1,2,3,6,12,40,98,229,540,1143,1989,2353,1629,660,213,59,18,6,2,1,0
Final size: 9005

Rep 9
1,2,6,14,33,91,249,581,1220,2127,2388,1464,550,158,58,21,7,2,0
Final size: 8972

Appendix B: More algorithms

Algorithm B1: Removing self-loops and parallel edges

This method removes self loops (edges with the same node as start and end) and multi-edges (e.g. pairs of edges which have identical starts and ends). Returns true on success, false if network could not be rewired.

```
bool Network::lose_loops() {
    if ( is_stopped() ) return false;
                                //all (outbound) edges in the network
    vector<Edge*> edges = get_edges();
    vector<Edge*>::iterator edge1, edge2;

    int m, n;
    int failed_attempts = 0;

    Node* start1;
    Node* start2;
    Node* end1;
    Node* end2;

    vector<Edge*> self_loops, multiedges;

    get_bad_edges( self_loops, multiedges);
    vector<Edge*> bad_edges;

    bad_edges.insert(bad_edges.begin(), self_loops.begin(), self_loops.end());
    bad_edges.insert(bad_edges.end(), multiedges.begin(), multiedges.end());

    //shuffle the vector
    int max = bad_edges.size() - 1;
    for (int i = max; i >= 0; i-- ) swap(bad_edges[i], bad_edges[ mtrand.randInt(i) ]);

    while ( bad_edges.size() > 0 ) {
        PROG( 50 + (int) (50 * (max - bad_edges.size()) / max) );
        m = bad_edges.size() - 1;
        n = mtrand.randInt(  edges.size() - 1 );
        if ( failed_attempts > 99 ) {
            cerr    << "It may be impossible to equilibriate a network with these parameters--"
                << "couldn't get rid of any self-loops or multi-edges in the last 100 attempts"
                << endl;
            return false;
        }
        if ( is_stopped() ) return false;

        Edge* edge1 = bad_edges[m];
        Edge* edge2 = edges[n];

        start1 = edge1->start;
        end1   = edge1->end;
        start2 = edge2->start;
        end2   = edge2->end;
        /*
        Rule 1: End of edge 1 must not equal start of edge 2, and vice versa.  At worst, this
prevents synonymous changes, and at best, it prevents some self-loops and multiedges.
```

```
        Rule 2: Start of edge 1 must not equal start of edge 2, and end of edge 1 must not equal
end of edge 2.  This prevents synonymous (i.e. non-structural, edge re-numbering) changes.

        Rule 3: End of edge 1 must not have an edge that goes to start of edge 2, and vice versa.
This may be expensive to enforce because it requires looking around two nodes for every proposed
change. It prevents some multiedges.
        */
        if ( end1 == start2  || end2 == start1 || start1 == start2 || end1 == end2 ) {
            failed_attempts++;
            continue;
        }

        // Rule 3
        vector<Node*> neighbors1 = end1->get_neighbors();
        vector<Node*> neighbors2 = end2->get_neighbors();
        vector<Node*>::iterator result1, result2;
        result1 = find( neighbors1.begin(), neighbors1.end(), start2 );
        result2 = find( neighbors2.begin(), neighbors2.end(), start1 );

        if( result1 != neighbors1.end() || result2 != neighbors2.end() ) {
            failed_attempts++;
            continue;
        }

        Edge* edge1_comp = edge1->get_complement();
        bad_edges.pop_back();

        for ( unsigned int i = 0; i < bad_edges.size(); i++ ) {
            if ( bad_edges[i]==edge1_comp ) {
                bad_edges.erase(bad_edges.begin() + i);
                break;
            }
        }
        failed_attempts = 0;
        edge1->swap_ends(edge2);
    }
    return true;
}
```

Algorithm B2: Percolation with cohorts

The percolation pseudocode in the main text cannot provide information about the epidemic curve as written. By separating infected individuals into cohorts (*new_infected* in the pseudocode below) we can extract more information about the epidemic without increasing the algorithmic complexity. Note that this algorithm is equivalent to a chain-binomial algorithm where the infectious period is fixed at 1.

```
Percolation(network, T):
   infected_queue ← empty list
   epidemic_curve ← empty list
   epidemic_size ← 0

   foreach node in network:
      set state of node to "susceptible"

   patient_zero ← random node from network
   set state of patient_zero to "infectious"
   append patient_zero to infected_queue
```

```
time ← 0
epidemic_curve[time] ← length of infected_queue

while infected_queue is not empty:
   time ← time + 1
   new_infected ← empty list
   foreach node in infected_queue:
      foreach neighbor of node:
         rand ← uniform random number between 0 and 1
         if neighbor is "susceptible"  and rand < T:
               set state of neighbor to "infectious"
               append neighbor to new_infected
      set state of node to "recovered"
      epidemic_size ← epidemic_size + 1
   infected_queue ← new_infected
   epidemic_curve[time] ← length of infected_queue
return epidemic_size
```

Algorithm B3: Alternative chain binomial

```
Chain_binomial(network, T, gamma):
   infected_queue ← empty list
   new_infected ← empty list
   foreach node in network:
      set state of node to "susceptible"
   patient_zero ← random node from network
   set state of patient_zero to "infected 1 day"
   append patient_zero to infected_queue
   while infected_queue is not empty:
      foreach node in infected_queue:
         foreach neighbor of node:
            rand ← uniform random number between 0 and 1
            if neighbor is "susceptible"  and rand < T:
                  set state of neighbor to "infected 1 day"
                  append neighbor to new_infected
         if node has been infected gamma days:
            remove node from infected_queue
            set state of node to "recovered"
         else:
            increment duration "infected" for node
      append new_infected to infected_queue
      new_infected ← empty list
   epidemic_size ← count of nodes in "recovered" state
   return epidemic_size
```
